# The GPR120 agonist TUG-891 mitigates ischemic brain injury by attenuating endoplasmic reticulum stress and apoptosis via the PI3K/AKT signaling pathway

**DOI:** 10.1016/j.neurot.2025.e00735

**Published:** 2025-09-06

**Authors:** Panxi Sun, Lili Wei, Xue Qin, Jia Luo, Dongsheng Fan, Yong Chen

**Affiliations:** a Department of Neurology, Peking University Third Hospital, Beijing, 100191, China; b Ministry-of-Education Key Laboratory of Xinjiang Endemic and Ethnic Diseases, School of Medicine, Shihezi University, Shihezi, 832000, China; c Beijing Key Laboratory of Biomarker and Translational Research in Neurodegenerative Diseases, Beijing, 100191, China; d Key Laboratory for Neuroscience, National Health Commission/Ministry of Education, Peking University, Beijing, 100191, China

**Keywords:** Ischemic stroke, TUG-891, GPR120, Endoplasmic reticulum stress, Apoptosis, PI3K/AKT signaling pathway

## Abstract

Extensive research has confirmed that omega-3 fatty acids provide cardiovascular protection primarily by activating the G protein-coupled receptor 120 (GPR120) signaling pathway. However, natural activators of this receptor often lack sufficient strength and precision. TUG-891, a recently synthesized selective GPR120 activator, has displayed significant therapeutic potential in multiple disease. This investigation seeks to evaluate the neuroprotective effects of TUG-891 against ischemic cerebral injury. To this end, an in vivo murine model of distal middle cerebral artery occlusion (dMCAO) was employed, alongside an in vitro model utilizing oxygen-glucose deprivation/reperfusion in HT22 ​cells. The results indicated that TUG-891 significantly enhanced neurological function, reduced the volume of cerebral infarction, and alleviated pathological damage following dMCAO. Moreover, TUG-891 demonstrated a significant reduction in oxidative stress levels, a decrease of markers related to endoplasmic reticulum (ER) stress, and the modulation of critical apoptotic regulators, thereby inhibiting apoptosis in both in vivo and in vitro settings. Additionally, TUG-891 was found to affect the PI3K/Akt signaling pathway, with the application of the inhibitor LY294002 negating the protective effects of TUG-891 in vitro. This comprehensive study reveals TUG-891's therapeutic potential for ischemic stroke through multi-target mechanisms involving oxidative stress mitigation, ER stress regulation, and survival pathway activation. The consistent neuroprotection observed across biological models underscores its translational value for further clinical development.

## Graphical abstract

**Figure:**
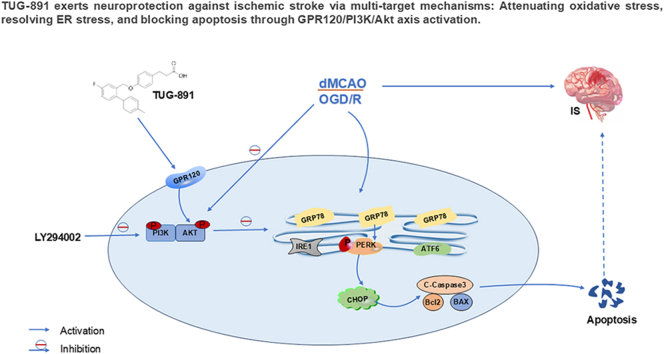

## Introduction

Stroke remains a major global health challenge, characterized by high incidence, disability, mortality, and substantial economic burden. According to the latest Global Burden of Disease 2021 data [1], stroke ranks as the third leading cause of death (7·3 million [95 ​% UI 6·6–7·8] deaths) and fourth leading cause of disability-adjusted life-year (DALY) (160·5 million [147·8–171·6] DALYs). In 2021, there were 93.8 million (89.0–99.3) prevalent cases of stroke and 11.9 million (10.7–13.2) incident strokes globally, with ischemic stroke accounting for 65.3 ​% (62.4–67.7) of all cases. Currently, tissue plasminogen activator is the main pharmacological intervention available for the treatment of ischemic stroke [2]. However, the clinical utility of tissue plasminogen activator remains hampered by a narrow temporal treatment window and safety concerns, posing significant barriers to broader clinical implementation. Consequently, there is an urgent imperative to identify and develop more effective therapeutic agents to enhance the clinical outcomes for stroke patients.

Epidemiological studies provide valuable insights for neuroprotective drug development. Omega-3 fatty acids—particularly eicosapentaenoic acid (EPA) and docosahexaenoic acid (DHA)—have attracted significant attention for their potential benefits in vascular diseases [3]. Population-based studies indicate that diets rich in marine-sourced omega-3 polyunsaturated fatty acids correlate with reduced susceptibility to atherosclerotic cardiovascular events and cerebrovascular accidents [4]. Higher omega-3 fatty acids levels are associated with lower risks of total and ischemic stroke [5] and DHA-containing plasma lipids show an inverse association with incident ischemic stroke [6]. Clinically, greater omega-3 intake correlates with reduced risk of fatal stroke [7]. However, clinical evidence for omega-3 benefits in cardiovascular disease remains inconsistent. A recent cardiovascular intervention trial demonstrated that marine-derived omega-3 supplementation provided no preventive benefit against cardiovascular complications in post-myocardial infarction patients aged ≥65 years [8]. In contrast, the landmark REDUCE-IT trial showed that high-dose EPA (4 ​g/day) significantly reduced composite cardiovascular outcomes [9]. This discrepancy may stem from dosage limitations: insufficient daily intake may lack efficacy, while higher doses (2–4 ​g/day) carry risks of contaminant exposure (e.g., heavy metals) and potential adverse effects like atrial fibrillation [10]. Thus, there is an urgent need for alternatives that activate downstream receptor for omega-3 fatty acids with high affinity at low concentrations to provide vascular disease protection.

Free fatty acid receptors (FFARs) are primarily responsible for recognizing different types of fatty acids, including long-chain, medium-chain, and short-chain fatty acids [11]. FFARs belong to the G protein-coupled receptor family and function by binding specific fatty acids to activate intracellular signaling cascades that modulate diverse physiological processes. Among the FFAR subtypes, G protein-coupled receptor 120 (GPR120, also referred to as FFAR4) exhibits particular sensitivity and serves as a principal receptor for omega-3 fatty acids [12]. GPR120 is predominantly expressed in the intestines, adipose tissue, pancreatic β-cells, and the nervous system. It plays a critical role in numerous cellular mechanisms and physiological functions, including neuroprotection, inhibition of cell proliferation, wound healing, appetite regulation, modulation of gut hormone secretion, lipid metabolism, anti-inflammatory responses, antidiabetic effects, and protection against atherosclerosis [11,13,14]. Given these properties—particularly its neuroprotective role—developing highly selective and potent GPR120 agonists represents a promising therapeutic strategy against ischemic stroke.

GPR120 agonist development is an active research focus, with several compounds now available [15]. TUG-891 is the first and most extensively studied GPR120 agonist, known for its high selectivity and activity [16]. It enhances lipid metabolism, reduces obesity [17], improves type 2 diabetes mellitus [18], ameliorates atherosclerosis [19], protects against acute kidney injury [20], alleviates nonalcoholic fatty liver disease [21], and inhibits lung adenocarcinoma [22]. Critically, recent studies show TUG-891 reduces neurological damage after cerebral hemorrhage by mitigating oxidative stress, endoplasmic reticulum (ER) stress, and apoptosis [23]. These findings suggest potential efficacy of TUG-891 in cerebral ischemic injury, which this study aims to investigate.

Concerning the underlying mechanisms, numerous prior functional investigations of TUG-891 have consistently demonstrated its anti-inflammatory, antioxidant, endoplasmic reticulum (ER) stress-alleviating, and anti-apoptotic properties mediated through activation of the GPR120 receptor. These mechanisms are also implicated in the pathophysiology of cerebral ischemic injury. Specifically, GPR120 is coupled to the Gq protein, and ligand-induced activation of GPR120 leads to an elevation in intracellular Ca^2+^ levels [11,15]. The resultant increase in intracellular Ca^2+^ concentration subsequently activates the phosphoinositide 3-kinase (PI3K) signaling pathway, including Akt, which promotes cell survival signaling [15]. Accordingly, we hypothesize that TUG-891 may attenuate ischemic neural damage by suppressing oxidative stress, ER stress and apoptosis via modulation of the PI3K/Akt signaling pathway.

## Materials and Methods

### Experimental animals

Male C57BL/6 mice, weighing between 20 and 23 ​g and aged 8–10 weeks, were obtained from the Animal Department of Peking University Health Science Center, operating under license number SYXK (Jing) 2022–0037. These animals were housed under standardized laboratory conditions with controlled temperature (23 ​± ​2)°C and humidity levels maintained at (60 ​± ​5)%, along with diurnal lighting cycles alternating every 12 ​h. Rodents received ad libitum access to conventional rodent chow and filtered water. All animal experiments complied with the ARRIVE guidelines and were performed in accordance with the U.K. Animals (Scientific Procedures) Act 1986, EU Directive 2010/63/EU, and the U.S. National Research Council's Guide for the Care and Use of Laboratory Animals. Experimental protocols were approved by the Institutional Animal Care and Use Committee of Peking University Health Sciences Center. Surgical interventions were conducted under anesthesia, and measures were implemented to minimize animal suffering.

### Permanent distal middle cerebral artery occlusion (dMCAO)

In this study, mice underwent permanent distal middle cerebral artery occlusion (dMCAO) to induce cerebral ischemia, following the methodology established by Doyle and Buckwalter [24]. Initially, the right common carotid artery was permanently ligated and sutured after the administration of anesthesia using 2.5 ​% tribromoethanol (0.16 ml/10 ​g), while maintaining the body temperature of the mice at 37 ​± ​0.5 ​°C. Subsequently, a 0.5-cm incision was made between the right lateral canthus and the right external auditory canal. The temporalis muscle was incised to expose the temporal bone, and the subcranial branches of the right medial cerebral cortex were examined microscopically. Through precise cranial osteotomy, microsurgical exposure of the right middle cerebral artery's cortical ramifications was achieved. The minimal aperture size ensured preservation of surrounding neurovascular structures while permitting necessary visualization. The middle cerebral artery was then occluded using an electrocoagulation pen. In the sham group, no ligation of the right common carotid artery was performed following dissection, and no cauterization was conducted after drilling to expose the middle cerebral artery.

### Experimental design

**Fig. 1:**
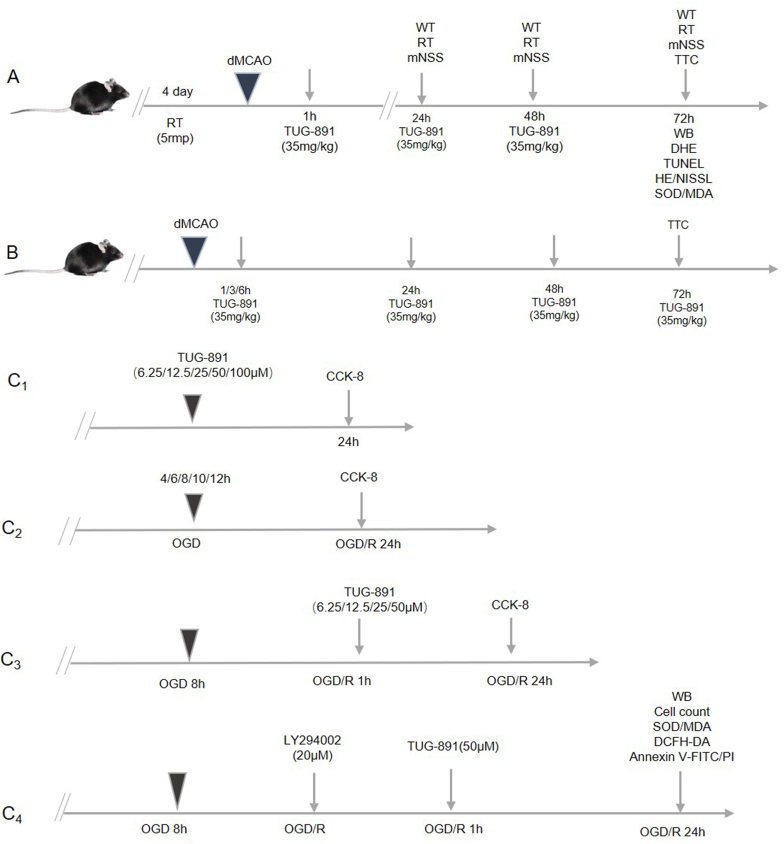
**Schematic representations of the experimental design.** This figure outlines the framework of in vivo and in vitro experiments to evaluate the neuroprotective effects of TUG-891. (A) Flowchart illustrating the in vivo experimental timeline for assessing TUG-891 administration (35 ​mg/kg, i.p.) at 1, 24, and 48 ​h post-dMCAO, with functional evaluations at 24, 48, and 72 ​h. (B) Experimental design to investigate time-dependent effects of TUG-891, with initial dosing at 1, 3, or 6 ​h post-dMCAO. (C1–C4) In vitro experimental setup for optimizing OGD duration and TUG-891 concentration, followed by mechanistic validation using the OGD/R model in HT22 ​cells with/without the PI3K inhibitor LY294002. **Key purpose:** To systematically assess the therapeutic window and cellular mechanisms of TUG-891 in ischemic injury. Abbreviations: OGD/R: Oxygen-glucose deprivation/reperfusion; dMCAO: distal middle cerebral artery occlusion; i.p.: intraperitoneal injection; h: hours.

The experimental design involved randomized allocation of mice into three distinct cohorts: sham-operated controls receiving mock procedures, cerebral ischemia models induced by dMCAO, and a therapeutic intervention cohort administered TUG-891 at a dosage regimen validated in prior preclinical investigations (35 ​mg/kg) [20,23]. TUG-891 (Catalog No: HY-100881, MCE, USA) was prepared in accordance with the manufacturer's instructions and administered via intraperitoneal injection at 1 ​h, 24 ​h, and 48 ​h post-modeling. The sham and dMCAO groups received an equivalent volume of saline as a control. Neurological deficit scores were assessed at 24 ​h, 48 ​h, and 72 ​h following the surgical procedure. Subsequent to the final evaluation of neurological scores, the mice were anesthetized and swiftly decapitated, allowing for the collection of brain tissue for further analysis (refer to Fig. 1A). To investigate the neuroprotective effects of delayed drug administration in the context of cerebral ischemia, the initial dose of TUG-891 was administered at predetermined time intervals (see Fig. 1B).

The cellular mechanism of TUG-891 was examined using an established OGD/R model in HT22 ​cells, replicating ischemia-reperfusion injury parameters in a cell culture system. Preliminary experiments were conducted to establish the optimal drug dosage and duration of OGD (illustrated in Fig. 1 C1–C3). In the formal experimental setup (depicted in Fig. 1 C4), the cells were categorized into four distinct groups: the control group, the OGD/R group, the OGD/R ​+ ​TUG-891 group, and the OGD/R ​+ ​TUG-891 ​+ ​LY294002 group (the latter serving as an inhibitor of the PI3K pathway).

### mNSS neurological score and rotarod test

The modified Neurological Severity Score (mNSS) is utilized primarily to evaluate motor function, sensory perception, balance, and reflexes in murine models. This scoring system ranges from 0 to 18, with elevated scores indicative of more severe neurological impairment. The mNSS behavioral assessment was conducted in a controlled, quiet environment at 24, 48, and 72 ​h post-surgery, with data meticulously recorded for each experimental group. Scoring was performed in a double-blinded fashion to ensure objectivity.

The Rotarod test was employed to assess motor coordination and fatigue resistance in the mice. Prior to the experimental modeling, the subjects underwent a training regimen at a speed of 5 ​revolutions per minute (RPM) for four consecutive days in a quiet setting. Only those mice that maintained their position on the apparatus for a minimum of 60 continuous seconds were selected for subsequent formal testing. During the test, the speed of the Rotarod was incrementally increased from 5 RPM to 40 RPM over a duration of 5 ​min. The time taken for each mouse to fall from the rod was recorded, and the results from three independent trials were averaged for each subject. Scoring for the Rotarod test was also conducted in a double-blinded manner to maintain the integrity of the data.

### 2,3,5-Triphenyltetrazolium chloride (TTC) staining and measurement of cerebral infarct size

Following a 72-h period, the brains were subjected to anesthesia using tribromoethanol, after which they were rapidly decapitated and subsequently frozen at −20 ​°C for a duration of 20 ​min. The cerebral specimens were subsequently processed by cutting them into coronally oriented sections measuring 2 ​mm thickness. These tissue sections were immediately transferred into a light-protected chamber containing a freshly prepared staining solution consisting of TTC at 2 ​% concentration (Solaibao Co., Beijing, China; Product ID T8170), where they underwent continuous immersion for 30 ​min. Throughout this staining procedure, environmental illumination was strictly controlled to maintain complete darkness. The size of the cerebral infarct was quantified using ImageJ software. The formula employed for calculating the brain tissue infarction rate was as follows: Brain tissue infarction rate (%) = (infarct size/total brain area) ​× ​100 ​%.

### Hematoxylin and Eosin (HE) staining and nissl staining

Mice were subjected to deep anesthesia and subsequently perfused via the left ventricle with saline followed by 4 ​% paraformaldehyde. The tissues were then fixed, dehydrated, embedded in paraffin, and sectioned for HE and Nissl staining. The staining procedures for HE (Catalog No: G1120, Beijing, Solaibao) and Nissl (Catalog No: G1430, Beijing, Solaibao) were conducted in accordance with the manufacturer's instructions for the staining solutions. Microscopic examination was performed to assess alterations in cortical neurons and Nissl bodies. For both staining assays, ten non-overlapping fields (at ​× ​400 magnification) in the ischemic penumbra were analyzed per tissue section and the number of HE-positive cells and Nissl-positive cells was counted in each field by using ImageJ software. All observers were blinded to group allocations to ensure objectivity.

### Measurement of malondialdehyde (MDA) and superoxide dismutase (SOD)Levels

Brain tissues that were harvested were homogenized in cold phosphate-buffered saline (PBS) and subsequently subjected to centrifugation at 12,000 ​g for 10 ​min to obtain the supernatants. Biochemical analysis of oxidative stress markers was performed using standardized protocols, with commercial assay kits (Beijing Solaibao, MDA: G1430; SOD: G1430) employed for spectrophotometric quantification of malondialdehyde and superoxide dismutase activities.

### Detection of reactive oxygen species (ROS) levels

ROS levels in animal tissues and cultured cells were evaluated using two distinct methodologies: dihydroethidium (DHE) immunofluorescence staining (Beijing Solaibao, Cat: G4817) and 2‘,7’-dichlorodihydrofluorescein diacetate (DCFH-DA) fluorescent probe labeling (Beijing Solaibao, Cat: CA1410). Following the experimental protocols specified in the reagent manuals, fluorescent signals were captured using a fluorescence microscope. Subsequent quantitative evaluation of fluorescence intensity was performed with ImageJ.

### TUNEL staining procedure

Murine brain specimens preserved in paraffin matrices underwent histological processing according to standardized protocols, with apoptotic cell identification being achieved through implementation of a commercial in situ labeling system (P-CA-301, Punosai Life Sciences, Wuhan). Upon completion of the staining process, apoptotic cells were visualized using a fluorescence microscope, and the analysis of apoptosis was conducted utilizing Image J software.

### Western blot

Total protein was extracted from mice brain tissue and cultured cells utilizing RIPA lysis buffer supplemented with PMSF and phosphatase inhibitors (Pulilai Company, China). The protein concentration was determined using a Protein BCA Kit (Pulilai Company, China). Subsequently, the proteins were subjected to electrophoresis, transferred to a membrane, and blocked. Following this, the membrane was incubated with primary antibodies (GPR120: Cat No. AF5219, America, Affinity; GPR78: Cat No. 11587-1-AP, Wuhan Protech; CHOP: Cat No. 66741-1-Ig, Wuhan Protech; PERK: Cat No. TP52759, Shanghai Abmart; P-PERK: Cat No. TA4499, Shanghai Abmart; P62: Cat No. T55546, Shanghai Abmart; LC3-II Cat No. T55992, Shanghai Abmart; GAPDH: Cat No. 60004-1-Ig, Wuhan Protech; β-Actin: Cat No. 66009-1-Ig, Wuhan Protech; Bax: Cat No. T40051, Shanghai Abmart; Bcl2: Cat No. T40056, Shanghai Abmart; Cleaved-caspase3: Cat No. 25128-1-AP, Wuhan Protech) and Horseradish peroxidase (HRP)-conjugated goat anti-rabbit and goat anti-mouse secondary antibodies (Cat No. SA00001-2, SA00001-1, Wuhan, Proteintech). The resulting signals were developed using a luminescent solution and quantified using Image J software prior to conducting statistical analysis with GraphPad software.

### Cell culture and administration

For in vitro investigations, HT22 neuronal cells were maintained in Dulbecco's Modified Eagle Medium containing 10 ​% fetal bovine serum under standard culture conditions (37 ​°C, 5 ​% CO_2_). Upon reaching approximately 80 ​% confluence, the cells were passaged, and those in the logarithmic growth phase were selected for subsequent experimental procedures.

To induce oxygen-glucose deprivation followed by OGD/R in HT22 ​cells, cells in the logarithmic growth phase were seeded in six-well plates and allowed to adhere overnight. After triple-rinsing with sterile PBS, the nutrient medium was exchanged for serum- and glucose-deficient medium. Cultures were then placed in a hypoxia-mimetic chamber (1 ​% O_2_,5 ​% CO_2_ and 94 ​% N_2_).Drug interventions were administered upon re-oxygenation, while the control group was maintained under normoxic conditions. Cell viability was evaluated using the Cell Counting Kit-8 (CCK-8) assay (Beyotime Biotechnology). To elucidate molecular mechanisms through pharmacological intervention, HT22 neuronal cultures were pharmacologically preconditioned with LY294002, a cell-permeable quinazolinone derivative with specific PI3K catalytic domain antagonism, at a concentration established in prior neuropharmacological protocols. This targeted pathway modulation preceded TUG-891 exposure following standardized preconditioning intervals.

### Flow cytometry experiment

The assessment of apoptosis in HT22 ​cells was conducted utilizing flow cytometry. Cells were initially seeded in six-well plates and cultured under standard conditions at 37 ​°C with 5 ​% CO2 for the control group. The treatment groups were maintained in a tri-gas controlled environment for an 8-h duration under OGD. Pharmacological intervention was subsequently initiated concurrent with atmospheric normalization. Subsequently, cells from each group were harvested and washed twice with pre-cooled PBS to achieve a concentration of approximately 1 x 10ˆ6 ​cells/ml. Following the protocol outlined in the Annexin V-FITC/PI double staining apoptosis detection kit, the subsequent procedures were executed. Ultimately, cell apoptosis was quantified using flow cytometry, and statistical analyses were performed.

### Statistical methods

Statistical analyses were conducted using GraphPad Prism software version 9.0.0 (GraphPad, San Diego, CA, USA). Results are reported as mean ​± ​SD except for the neurological scores (mNSS), where median ​± ​range was used. Shapiro-Wilk test was used to check normality, and Bartlett's test was used to check equal variance. The statistical significance was assessed using either unpaired *t*-test or one-way ANOVA followed by Dunnett's multiple comparisons test or two-way ANOVA followed by Holm-Sidak's multiple comparisons test (for normally distributed data), and Mann Whitney test (for not normally distributed data). P ​< ​0.05 was considered to be statistically significant.

## Results

### Post-treatment with TUG-891 enhances the GPR120 levels and mitigates cerebral damage in mice subjected to dMCAO

**Fig. 2:**
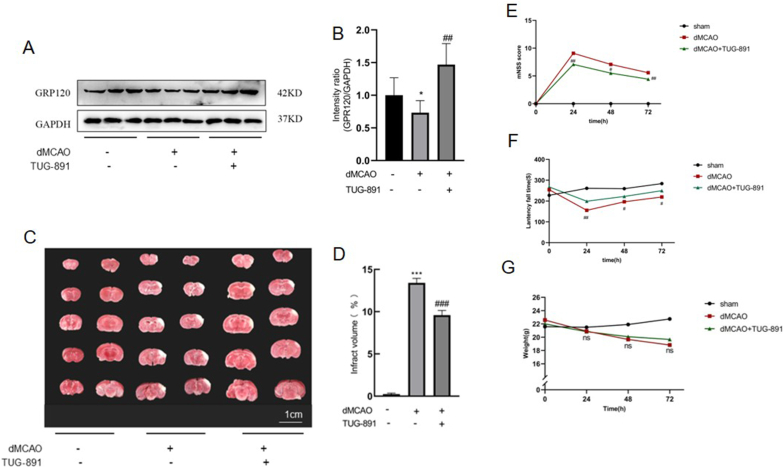
**Post-treatment with TUG-891 enhances GPR120 levels and mitigates cerebral damage in mice subjected to dMCAO.** (A) Representative Western blot images of GPR120; (B) Quantitative analysis showing reduced GPR120 in the dMCAO group and upregulation by TUG-891. (C) Representative TTC-stained coronal sections (viable tissue: red; infarct: pale); (D) Quantification demonstrating TUG-891 significantly reduced infarct volume at 72 ​h. (E) mNSS scores indicating improved neurological function in TUG-891-treated mice. (F) Rotarod test results showing increased latency to fall (enhanced motor coordination) with TUG-891. (G) No significant body weight differences among groups. **Key findings:** TUG-891 upregulates GPR120, reduces infarct size, and improves neurological and motor function post-ischemia. Data are mean ​± ​SD or median ​± ​range for mNSS (WB/TTC: N ​= ​5; functional tests: N ​= ​12). Statistical significance: ∗P ​< ​0.05, ∗∗∗P ​< ​0.001 vs. sham; #P ​< ​0.05, ##P ​< ​0.01, ###P ​< ​0.001 vs. dMCAO. Abbreviations: WB: Western Blot; TTC: 2,3,5-Triphenyltetrazolium chloride; SD: Standard deviation; mNSS: Modified Neurological Severity Score.

To assess the effect of GPR120 agonist TUG-891 in modulating the GPR120 levels in ischemic stroke, mice underwent a procedure of dMCAO, followed by Western blot analysis to evaluate the GPR120 levels. The findings revealed the GPR120 levels reduced within the ischemic cortex in the dMCAO group when compared to the control group (see Fig. 2A–B). Furthermore, as illustrated in Fig. 2A–B, TUG-891 treatment resulted in a significant increase in GPR120 expression levels in the TUG-891 group relative to the dMCAO group. These results prove that GPR120 takes part in the pathological processes associated with cerebral ischemia and that TUG-891 has the capacity to modulate the expression of GPR120.

In order to examine the effects of TUG-891 on ischemic brain injury, a model of distal dMCAO was utilized in murine subjects. TUG-891 was administered at multiple time intervals, as depicted in Fig. 1A. The extent of cerebral infarction was evaluated using TTC staining. Mice subjected to the dMCAO procedure exhibited substantial infarcts in the cortical regions of the brain (Fig. 2C). Notably, post-treatment with TUG-891 led to a significant reduction in the areas of cerebral infarction when compared to the dMCAO group (Fig. 2D). Furthermore, TUG-891 post-treatment was associated with a marked decrease in the modified mNSS (Fig. 2E) and an increase in latency fall time (Fig. 2F) relative to the dMCAO group. However, no significant differences in body weight were noted between the two groups (Fig. 2G). Collectively, these results indicate that post-treatment with TUG-891 effectively diminished infarct volume and enhanced motor function recovery in mice subjected to dMCAO.

**Supplemental Figure 1:**
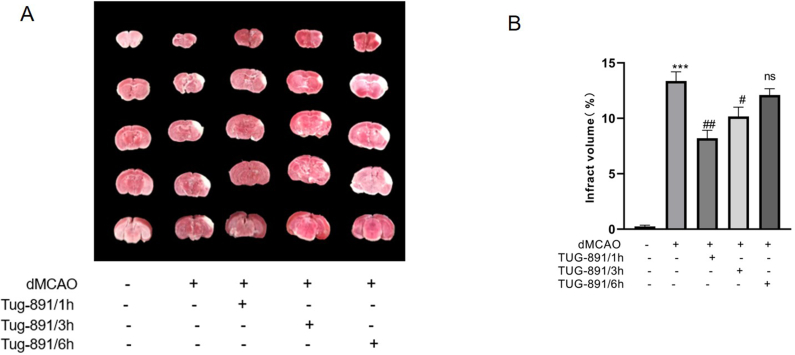
TUG-891 reduces brain tissue injury in mice subjected to dMCAO in a time-dependent manner. (A) Representative TTC-stained sections showing infarct size variation with TUG-891 dosing time. (B) Quantification indicating significant infarct reduction with 1-h and 3-h dosing, but not 6-h dosing. **Key finding:** TUG-891's neuroprotective effect is time-dependent, with optimal efficacy when administered within 3 ​h post-ischemia. Data are mean ​± ​SD (N ​= ​6). Statistical significance: ∗P ​< ​0.05, ∗∗P ​< ​0.01 vs. sham.

In clinical practice, stroke patients are typically candidates for therapeutic interventions within a few hours following arterial occlusion. Therefore, we assessed the effects of delayed treatment with TUG-891. The initial dose of TUG-891 was administered at designated time intervals (1 ​h, 3 ​h, or 6 ​h post-operation). Our results indicated that the most favorable outcomes were achieved with administration at 1 ​h post-operation (refer to Supplementary Fig. 1A and B), while treatment at 3 ​h post-operation also resulted in a significant reduction in infarct volume. In contrast, treatment administered at 6 ​h post-operation did not exhibit any protective effects.

### TUG-891 administration improves histopathological changes and modulate oxidative stress levels in ischemic brain after dMCAO

**Fig. 3:**
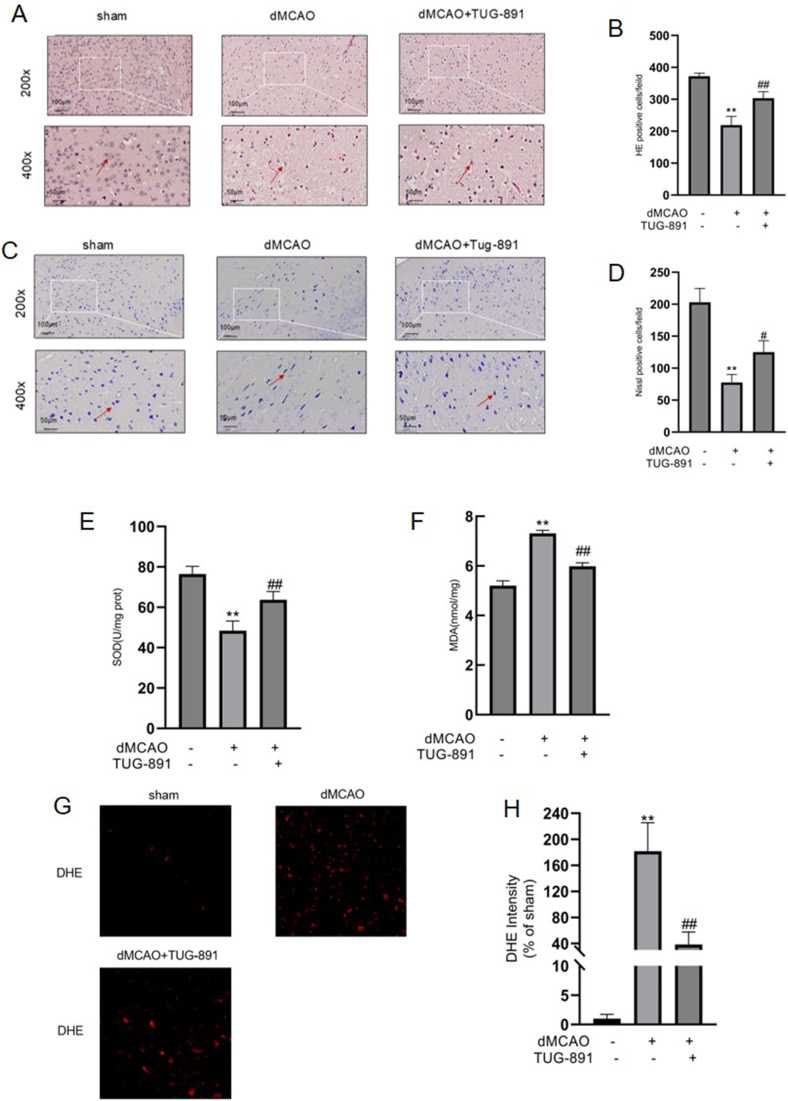
**TUG-891 administration improves histopathological changes and reduces oxidative stress levels in ischemic brain after dMCAO.** (A) HE staining showing disorganized neurons with pyknotic nuclei in dMCAO mice, ameliorated by TUG-891 ( ​× ​200, ​× ​400); (B) HE positive cell count quantification confirming TUG-891's protective effect. (C) Nissl staining demonstrating preserved Nissl bodies in the penumbra with TUG-891 treatment ( ​× ​200, ​× ​400); (D) Nissl positive cell quantification confirming TUG-891's protective effect. (E–F) Biochemical analysis showing TUG-891 restored SOD levels (E) and reduced MDA levels (F). (G–H) DHE fluorescence images (G) and quantification (H) indicating decreased ROS production in TUG-891-treated mice. **Key findings:** TUG-891 alleviates neuronal damage and mitigates oxidative stress (reduced ROS/MDA, increased SOD) post-ischemia. Data are mean ​± ​SD (N ​= ​5). Statistical significance: ∗∗P ​< ​0.01 vs. sham; ##P ​< ​0.01 vs. dMCAO. Abbreviations: HE: Hematoxylin and Eosin staining; SOD: Superoxide dismutase; MDA: Malondialdehyde; ROS: Reactive oxygen species; DHE: Dihydroethidium.

To assess the therapeutic potential of TUG-891 in alleviating neuronal damage caused by dMCAO within murine ischemic brain, HE and Nissl staining techniques were employed for histopathological evaluation. The HE staining results indicated that neural cells within the ischemic cortex of the dMCAO group exhibited significant damage, characterized by disorganized neuronal architecture and a marked presence of pyknotic nuclei (Fig. 3A). Following treatment with TUG-891, there was a notable reduction in dMCAO-induced neural injury (Fig. 3A). Quantitative analysis revealed a significant reduction in HE-positive cells following cerebral ischemia, whereas TUG-891 treatment markedly increased neuronal survival (Fig. 3B). Similarly, Nissl staining revealed that TUG-891 treatment led to a decrease in damage to Nissl bodies in the penumbral area (Fig. 3C–D). Collectively, these findings suggest that TUG-891 has the potential to mitigate neural injury resulting from dMCAO.

To evaluate the therapeutic potential of TUG-891 against redox imbalance in cerebral ischemic injury, we conducted systematic quantification of oxidative stress biomarkers in cerebral tissue lysates via enzymatic activity profiling (SOD) and lipid peroxidation product detection (MDA). Comparative biochemical analysis revealed distinct pathological alterations in dMCAO models relative to surgical controls, characterized by SOD depletion and MDA accumulation in ischemic cortical regions (Fig. 3E–F). Pharmacological intervention with TUG-891 elicited a restorative redox response, demonstrating cortical SOD restoration and concurrent MDA attenuation relative to untreated counterparts (Fig. 3E–F). The dMCAO intervention triggered a distinct oxidative microenvironment, as evidenced by DHE-derived fluorescence imaging showing elevated oxidative species burden in ischemic regions compared to baseline physiological conditions. Notably, this oxidative stress response was substantially attenuated following TUG-891 administration, as evidenced by reduced fluorescence intensity measurements(Fig. 3G–H).These findings suggest that TUG-891 may alleviate ischemic neuronal damage through the modulation of oxidative stress.

### TUG-891 inhibits neuronal apoptosis in dMCAO mice

**Fig. 4:**
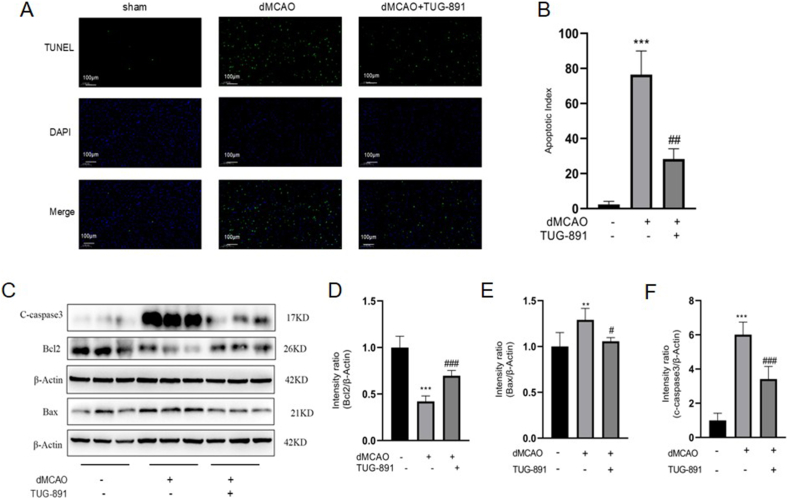
**TUG-891 inhibits neuronal apoptosis in dMCAO mice.** (A–B) TUNEL staining images (A) and quantification (B) showing fewer apoptotic cells in TUG-891-treated mice. (C) Representative western blots of apoptosis-related proteins; (D–F) Quantitative analysis indicating TUG-891 upregulated anti-apoptotic Bcl-2 (D) and downregulated pro-apoptotic Bax (E) and Cleaved Caspase-3 (F). **Key findings:** TUG-891 reduces ischemic neuronal apoptosis by modulating the Bcl-2/Bax axis and inhibiting Caspase-3 activation. Data are mean ​± ​SD (N ​= ​5). Statistical significance: ∗∗P ​< ​0.01, ∗∗∗P ​< ​0.001 vs. sham; #P ​< ​0.05, ##P ​< ​0.01, ###P ​< ​0.001 vs. dMCAO. Abbreviations: Bcl-2: B-cell lymphoma-2; Bax: BCL-2-associated X protein; TUNEL: Terminal deoxynucleotidyl transferase dUTP nick end labeling.

To assess the impact of TUG-891 on neuronal apoptosis in dMCAO mice, apoptotic cells within the cortical regions were identified using TUNEL staining. The experimental data demonstrated pronounced elevation of programmed cell death events in dMCAO cohorts compared to non-ischemic controls (Fig. 4A–B). Pharmacological intervention with TUG-891 exhibited substantial attenuation of apoptotic bodies formation relative to untreated ischemic models (Fig. 4A–B).

Furthermore, western blotting assay was conducted to evaluate the expression levels of apoptosis-related proteins in the ischemic region. Immunoblotting analysis demonstrated distinct apoptotic regulatory profiles between experimental groups. Investigative profiling identified differential molecular signatures between ischemic and sham-operated neural tissues. Cerebral specimens subjected to dMCAO intervention exhibited coordinated downregulation of anti-apoptotic Bcl-2 alongside pronounced accumulation of pro-apoptotic effector Bax and activated caspase-3 fragments, indicative of apoptosis pathway activation (Fig. 4C–F).Notably, pharmacological intervention with TUG-891 effectively reversed these pathological alterations, demonstrating enhanced Bcl-2 expression concurrent with substantial suppression of both Bax accumulation and Caspase-3 activation compared to untreated ischemic models (Fig. 4C–F). These results suggest that TUG-891 effectively mitigates apoptosis induced by dMCAO.

### TUG-891 alleviates ER stress in dMCAO mice

**Fig. 5:**
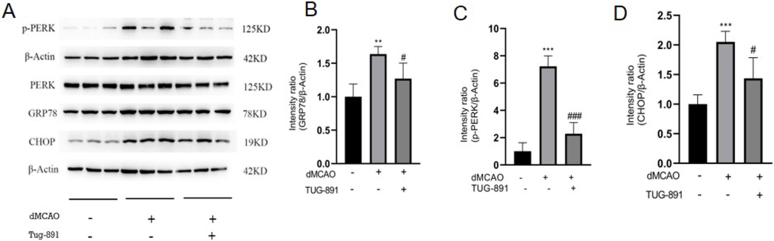
**TUG-891 alleviates ER stress in mice following dMCAO injury.** (A) Representative western blots of ER stress markers; (B–D) Quantitative analysis showing TUG-891 reduced expression of GRP78 (B), phosphorylated PERK (P-PERK, C), and CHOP (D) compared to the dMCAO group. **Key findings:** TUG-891 suppresses ischemia-induced ER stress by downregulating critical markers of the PERK-CHOP pathway. Data are mean ​± ​SD (N ​= ​5). Statistical significance: ∗∗P ​< ​0.01, ∗∗∗P ​< ​0.001 vs. sham; #P ​< ​0.05, ###P ​< ​0.001 vs. dMCAO. Abbreviations: ER: Endoplasmic reticulum; GRP78: Glucose-regulated protein 78kD; CHOP: C/EBP-homologous protein; P-PERK: Phosphorylated protein kinase RNA-like endoplasmic reticulum kinase.

ER stress play a crucial role in the apoptotic cascade. To elucidate the mechanistic connection between attenuated neuronal apoptosis and ER stress modulation, comprehensive profiling of ER stress-related biomarkers was performed. Biochemical assessments demonstrated coordinated activation of the ER stress cascade, evidenced by marked elevation in GRP78 expression, enhanced PERK phosphorylation, and pronounced CHOP upregulation in cerebral ischemia models compared with sham (Fig. 5A–D). Pharmacological intervention with TUG-891 exhibited marked attenuation of ER stress signaling pathways within the ischemic brain (Fig. 5A–D). These results suggest that TUG-891 may exert its protective effects against neuronal apoptosis through the modulation of ER stress pathways.

### TUG-891 mitigates neuronal damage in vitro following OGD/R through PI3K/Akt signaling pathway

**Fig. 6:**
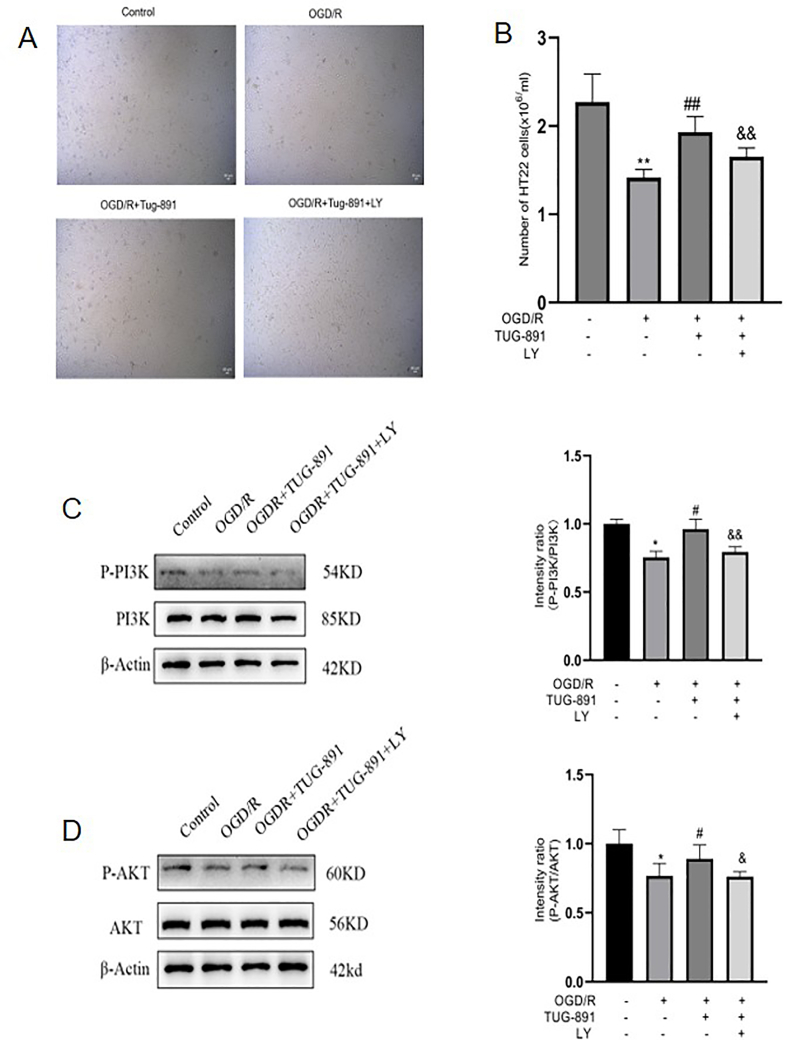
**TUG-891 mitigates neuronal damage in vitro following OGD/R through the PI3K/Akt signaling pathway.** (A) Representative images of HT22 ​cells showing reduced viability in OGD/R, rescued by TUG-891 (reversed by LY294002). (B) Cell count quantification confirming TUG-891's protective effect. (C–D) Western blot analysis demonstrating TUG-891 upregulated *p*-AKT (C) and p-PI3K (D), effects abolished by LY294002. **Key findings:** TUG-891 protects HT22 ​cells from OGD/R injury by activating the PI3K/Akt pathway. Data are mean ​± ​SD (N ​= ​3 or 5). Statistical significance: ∗P ​< ​0.05, ∗∗P ​< ​0.01 vs. control; #P ​< ​0.05, ##P ​< ​0.01 vs. OGD/R; &P ​< ​0.05, &&P ​< ​0.01 vs. OGD/R ​+ ​TUG-891. Abbreviations: *p*-AKT: Phosphorylated AKT protein; p-PI3K: Phosphorylated phosphoinositide 3-kinase; LY294002: PI3K inhibitor.

**Supplemental Figure 2:**
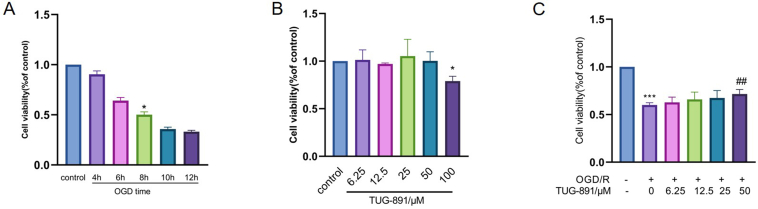
TUG-891 inhibits cell death after OGD/R injury: optimization of OGD duration and drug concentration. (A) CCK8 assay showing 8-hour OGD induced significant HT22 cell death (optimal duration). (B) TUG-891 (50 μM) had no toxic effect on normal HT22 cells. (C) CCK8 quantification confirming 50 μM TUG-891 as the optimal concentration for protecting OGD/R-exposed HT22 cells. **Key findings:** 8-hour OGD and 50 μM TUG-891 were selected for subsequent in vitro experiments. Data are mean±SD (N=5). Statistical significance: ∗P<0.05, ∗∗∗P<0.001 vs. control; ##P<0.01 vs. OGD/R. Abbreviations: CCK8: Cell Counting Kit-8.

Therapeutic intervention with TUG-891 was subsequently subjected to rigorous pharmacological profiling in an established OGD/R paradigm, focusing on its capacity to mitigate ischemia-reperfusion associated cytopathology through modulation of cellular stress pathways. HT22 cells were exposed to oxygen-glucose deprivation for varying durations, and cell viability was measured using the CCK8 assay. The optimal duration for OGD was established as 8 ​h (see Supplemental Fig. 2A). A systematic evaluation was conducted to assess the dose-dependent neuroprotective efficacy of TUG-891 on HT22 neuronal cultures under both basal physiological states and ischemic-reperfusion injury paradigms. Through experimental validation, the optimal cytoprotective dosage was identified as 50 ​mM (Supplementary Fig. 2B and C). Following the OGD/R stimulation, there was a significant reduction in the number of cells when compared to the control group that did not experience OGD/R. However, the administration of 50 ​mM of TUG-891 led to an increase in cell viability, indicating a protective effect against the injury induced by OGD/R (see Fig. 6A–B). In contrast, following the LY294002 treatment, the status and number of HT22 ​cells did not differ significantly from those in the OGD/R group (see Fig. 6A–B).

To further investigate the involvement of the PI3K/AKT signaling pathway in the neuroprotective effects of TUG-891, we examined the expression of proteins associated with this pathway. Our results indicated that OGD/R led to a reduction in the phosphorylation of PI3K and AKT (see Fig. 6C–D). Conversely, post-treatment with TUG-891 significantly enhanced the phosphorylation of both PI3K and AKT, an effect that was negated by LY294002 (see Fig. 6C–D). The results indicate that the neuroprotective properties of TUG-891 may, at least in part, be facilitated by the modulation of the PI3K/AKT signaling pathway.

### TUG-891 inhibits neuronal apoptosis in vitro following OGD/R through PI3K/Akt signaling pathway

**Fig. 7:**
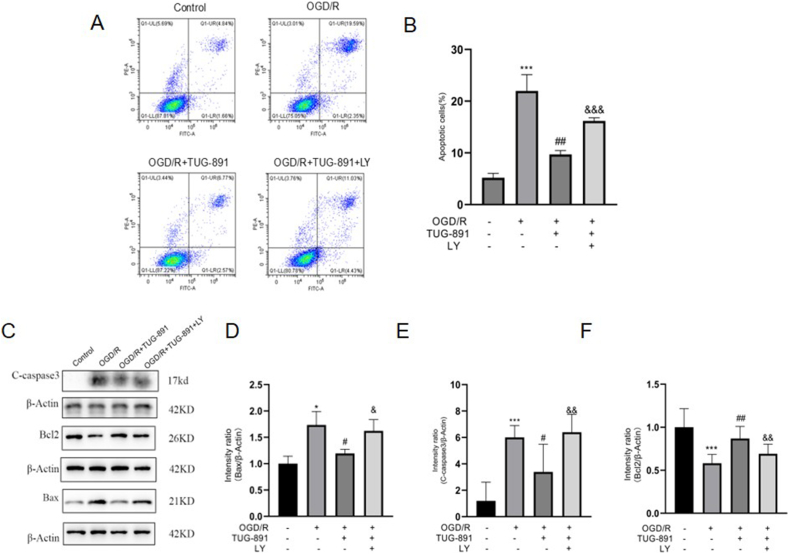
**TUG-891 inhibits neuronal apoptosis in HT22 ​cells exposed to OGD/R via the PI3K/Akt signaling pathway.** (A–B) Flow cytometry analysis showing TUG-891 reduced OGD/R-induced apoptosis, reversed by LY294002. (C) Representative western blots of apoptotic proteins; (D–F) Quantification indicating TUG-891 downregulated Bax (D) and Cleaved Caspase-3 (E), and upregulated Bcl-2 (F) (effects blocked by LY294002). **Key findings:** TUG-891 inhibits OGD/R-induced apoptosis in HT22 ​cells through PI3K/Akt-dependent modulation of apoptotic proteins. Data are mean ​± ​SD (N ​= ​5). Statistical significance: ∗P ​< ​0.05, ∗∗∗P ​< ​0.001 vs. control; #P ​< ​0.05, ##P ​< ​0.01 vs. OGD/R; &P ​< ​0.05, &&P ​< ​0.01, &&&P ​< ​0.001 vs. OGD/R ​+ ​TUG-891.

The impact of TUG-891 on apoptosis in HT22 ​cells was examined through the application of Annexin V-FITC/PI double staining flow cytometry. The results revealed that the control group exhibited a low incidence of apoptosis, whereas the OGD/R group showed a significant increase in apoptosis levels relative to the control group (Fig. 7A–B). Importantly, treatment with TUG-891 resulted in a substantial reduction in apoptosis among HT22 ​cells (Fig. 7A–B). Furthermore, LY294002 treatment attenuated the protective effect of TUG-891.

In addition, through Western blot experimentation, we investigated the apoptotic status of HT22 ​cells at the protein level. We found that compared to the OGD/R group, TUG-891 was able to decrease the level of the pro-apoptotic protein BAX and cleaved-caspase3 (Fig. 7C–E) and significantly elevate the level of the anti-apoptotic protein Bcl-2 (Fig. 7F), with statistical significance. However, this protective effect of TUG-891 could be attenuated or counteracted by the pre-treatment with inhibitors of the PI3K/AKT pathway.

### TUG-891 regulates oxidative stress and ER stress in HT22 ​cells after OGD/R injury via the PI3K/Akt signaling pathway

**Fig. 8:**
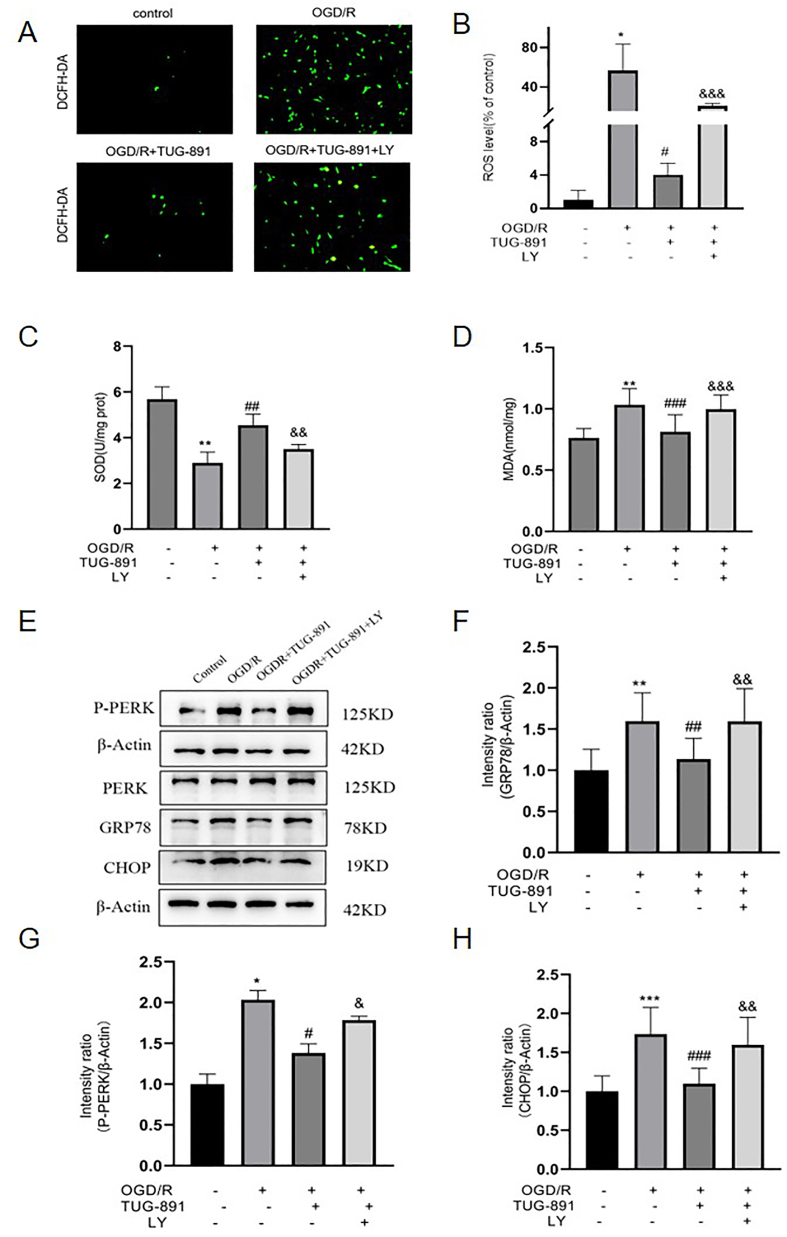
**TUG-891 reduces oxidative stress and ER stress in HT22 ​cells after OGD/R injury via the PI3K/Akt signaling pathway.** (A–B) DCFH-DA fluorescence showing TUG-891 reduced ROS, reversed by LY294002. (C–D) Biochemical analysis demonstrating TUG-891 increased SOD (C) and decreased MDA (D) (effects abolished by LY294002). (E–H) Western blots and quantification showing TUG-891 downregulated GRP78 (F), P-PERK (G), and CHOP (H), reversed by LY294002. **Key findings:** TUG-891 alleviates OGD/R-induced oxidative stress and ER stress in HT22 ​cells via PI3K/Akt activation. Data are mean ​± ​SD (N ​= ​5). Statistical significance: ∗P ​< ​0.05, ∗∗P ​< ​0.01, ∗∗∗P ​< ​0.001 vs. control; #P ​< ​0.05, ##P ​< ​0.01, ###P ​< ​0.001 vs. OGD/R; &P ​< ​0.05, &&P ​< ​0.01, &&&P ​< ​0.001 vs. OGD/R ​+ ​TUG-891. Abbreviations: DCFH-DA: 2′,7′-Dichlorofluorescin diacetate (ROS probe).

To assess the influence of TUG-891 on oxidative stress levels in vitro, HT22 ​cells were subjected to OGD/R with or without TUG-891 and LY294002. The OGD/R intervention induced a specific oxidative phenomenon, as demonstrated by DCFH-DA fluorescence imaging, which revealed an increased burden of oxidative species in the OGD/R group relative to the control group. Importantly, the oxidative stress response was significantly diminished following the administration of TUG-891, as indicated by decreased fluorescence intensity measurements (Fig. 8A–B). However, the effects of TUG-891 were completely negated by treatment with LY294002.Oxidative stress makers SOD and MDA levels were also measured as described. The result revealed that the OGD/R stimulation exhibited a significant decrease in SOD content and a significant increase in MDA content (Fig. 8C–D). Furthermore, compared to the OGD/R group, stimulation with TUG-891 resulted in a decrease in SOD content and a significant reduction in MDA levels (Fig. 8C–D). However, the effect of Tug-891 were abolished by treatment with LY294002.These results indicate that TUG-891 can alleviate oxidative stress induced by OGD/R at the cellular level.

The effect of TUG-891 on ER stress in HT22 ​cells after OGD/R injury was also examined in vitro. As illustrated in Fig. 8E-H, OGD/R stimulation resulted in a significant increase in the levels of ER stress markers (GRP78, *P*-PERK, CHOP), when compared with control group. Furthermore, pharmacological intervention with TUG-891 demonstrated a notable reduction in the activation of ER stress signaling pathways. However, the beneficial effects of TUG-891 on ER stress were diminished in the presence of the PI3K/AKT pathway inhibitor LY294002. These results indicate that TUG-891 not only mitigates ER stress but may also be linked to the PI3K/AKT signaling pathway.

## Discussion

Researches have indicated that omega-3 fatty acids, specifically EPA and DHA, confer benefits to cardiovascular health. A substantial body of literature has demonstrated that EPA and DHA offer protective effects against cerebral ischemic injury [[25], [26], [27]]. Therapeutic nutritional frameworks now emphasize systematic integration of long-chain polyunsaturated fatty acids into daily dietary patterns, particularly targeting primary prevention of cardiovascular disease. However, it is noteworthy that not all clinical trials have corroborated the beneficial effects of omega-3 fatty acids. This raises the possibility that synthetic analogues with a high affinity for receptors at low concentrations may provide enhanced efficacy in the protection against vascular diseases. GPR120 (FFAR4) and GPR40 (FFAR1) both function as receptors for omega-3 fatty acids; however, GPR120 exhibits significantly higher affinity for long-chain polyunsaturated omega-3 fatty acids (e.g., DHA and EPA), while GPR40 is preferentially activated by saturated long-chain fatty acids such as palmitic acid [28]. Although preclinical studies indicate that the GPR40 agonist LY2922470 mitigates acute brain injury [29], clinical investigations have revealed dose-limiting hepatotoxicity associated with GPR40-targeted therapies [30]. Given these safety concerns surrounding GPR40 activation and the limited exploration of GPR120 modulators in cerebral ischemia, this study focuses on evaluating the neuroprotective efficacy of TUG-891—a potent and selective GPR120 agonist—against ischemic brain injury. Notably, preclinical evidence indicates that TUG-891 not only lacks significant adverse effects but conversely demonstrates protective effects on hepatic and renal function [[19], [20], [21],^31^]. This study represents the inaugural investigation demonstrating that the synthetic GPR120 agonist provides a protective effect against cerebral ischemia. The findings indicate that post-treatment with TUG-891 significantly diminished cerebral infarct size, mitigated neurological deficits, and reduced neuronal loss.

Previous studies have been proved that GRP120 may play a role in the cerebral ischemic injury process and activating GPR120 by DHA or EPA could migrate neuronal injury during the ischemic events [26,27]. However, specificity of these naturally occurring GPR120 agonists is constrained, as they also interact with GPR40 and other receptors [32,33]. Our findings utilizing the synthetic GPR120 agonist TUG-891 further substantiate that the selective activation of GPR120 can confer neuroprotective effects. Moreover, given that neuroprotective therapies are typically initiated post-onset of ischemic stroke in clinical settings, experimental studies that administer drugs after the onset of ischemia are of greater significance for clinical trials. In contrast to previous studies that involved pre-treatment with drugs, the administration of TUG-891 3 ​h after the onset of stroke in the current study still demonstrated neuroprotective properties, thereby enhancing its clinical relevance.

In the present study, we observed decreased GPR120 expression in mice at day 3 post-ischemic injury, implicating its involvement in cerebral ischemic pathophysiology—consistent with prior report [14]. TUG-891 intervention restored GPR120 protein levels, confirming neuroprotection via GPR120 activation. Previous investigations have established that GPR120 activation triggers cytoprotective responses through downstream effectors (Gq, Gs, Gi, β-arrestin) [34]. Crucially, Gq coupling directly activates PI3K/Akt signaling and/or elevates intracellular Ca^2+^, which further potentiates PI3K/Akt activation [15]. This pathway serves as a master regulator of cell survival, metabolism, and apoptosis suppression, with established neuroprotective roles in cerebral ischemia [35]. Activated PI3K/Akt modulates key injury mechanisms: oxidative stress, endoplasmic reticulum stress (ERS), and neuronal apoptosis [36]. In this study, we found that ischemic hypoxic injury led to decreased levels of p-PI3K) and *p*-Akt, indicating that the PI3K/Akt signaling pathway was inhibited during ischemic/hypoxic injury, consistent with previous findings. Treatment with TUG-891 elevated p-PI3K and *p*-Akt expression, an effect that was abrogated by the PI3K/Akt pathway inhibitor LY294002. Collectively, these data demonstrate TUG-891 exerts neuroprotection by activating GPR120 and its downstream PI3K/Akt axis.

Oxidative stress reflects a pathological imbalance between pro-oxidant forces and antioxidant defenses. In acute ischemic stroke, this imbalance drives tissue damage via protein misfolding, mitochondrial dysfunction, and lipid peroxidation [37]. The PI3K/Akt pathway counters oxidative injury by activating transcription factors (e.g., Nrf2, FOXO3a), elevating antioxidants like SOD while suppressing ROS and MDA(35).In this study, both animal and cellular models confirmed that ischemia-hypoxia injury leads to abnormal oxidative stress levels, characterized by increased ROS and MDA levels and decreased levels of the antioxidant SOD. TUG-891 reversed these changes in vivo and in vitro models. Critically, LY294002 (PI3K/Akt inhibitor) abolished TUG-891's effects in cellular assays. These findings establish TUG-891 mitigates oxidative stress through GPR120/PI3K/Akt activation, defining a neuroprotective mechanism.

Cerebral ischemic injury triggers irreversible neuronal loss through necrosis and apoptosis. While apoptosis involves complex regulatory cascades, it presents a tractable therapeutic target [38]. Ischemia-induced oxidative/ER stress, inflammation, and excitotoxicity converge to activate caspase-dependent apoptosis. This process is regulated by Bcl-2 family proteins—where Bcl-2 inhibits and Bax promotes apoptosis—culminating in caspase-3 cleavage to its active form (cleaved caspase-3), the executioner protease of cell death [39]. The PI3K/AKT pathway counters ischemic apoptosis by phosphorylating downstream targets (e.g., mTOR, NF-κB, GSK-3β). It mediates neuroprotection by suppressing pro-apoptotic effectors (Bax, caspase-3) while enhancing anti-apoptotic Bcl-2(35). In this study, we confirmed through TUNEL and flow cytometry techniques that ischemic injury promotes cell apoptosis, while TUG-891 can reduce apoptosis. At the molecular level, we further demonstrated that TUG-891 upregulates the anti-apoptotic protein Bcl-2 and inhibits the levels of pro-apoptotic proteins Bax and cleaved caspase-3. The use of the PI3K/Akt pathway inhibitor LY294002 reversed the anti-apoptotic effects of TUG-891. These findings establish TUG-891 mitigates neuronal apoptosis via GPR120/PI3K/Akt activation, defining a core neuroprotective mechanism.

Cerebral ischemia-induced ER stress significantly contributes to ischemic injury, representing a key therapeutic target [40]. Ischemic disruption of ER homeostasis activates the PERK pathway: under physiological conditions, PERK associates with GRP78 maintaining inactivity, but ischemia triggers GRP78 dissociation. This activates PERK signaling, initiating a protective unfolded protein response. Sustained activation however upregulates pro-apoptotic effector CHOP, driving neuronal death. Previous studies indicate PI3K/Akt activation could mitigate ER stress by suppressing GRP78, PERK phosphorylation, and CHOP expression [41]. In this study, both in vivo and in vitro experiments demonstrated that ischemic injury induces an ER stress response, as evidenced by increased expression levels of ER stress marker proteins GRP78, P-PERK, and CHOP, which is consistent with previous research. Treatment with TUG-891 significantly suppressed ER stress, shown by decreased expression levels of GRP78, P-PERK, and CHOP. Furthermore, in vitro experiments confirmed that the PI3K/Akt pathway inhibitor LY294002 could reverse the effects of TUG-891. These results indicate that TUG-891 may alleviate ER stress and exert neuroprotective effects through the GPR120/PI3K/Akt pathway.

While this investigation highlights the preclinical neuroprotection potential of TUG-891, several limitations warrant consideration. First, we only conducted experiments using the dMCAO model in mice; future research needs to validate the findings in other models, as well as in other animals or non-human primates. Second, the dosing regimen in this study—TUG-891 administration at 35 ​mg/kg at 1, 3, and 6 ​h post-ischemia for a 3-day treatment course—was selected based on established preclinical protocols [20,23]. However, further investigations are warranted to optimize dosing parameters (including long-term safety profiles, potential adverse effects, and ideal therapeutic windows), particularly for extended treatment durations. Third, this study confirmed that TUG-891 exerts neuroprotective effects through the GPR120/PI3K/Akt pathway. However, we did not further explore how TUG-891 affects downstream targets of the PI3K/Akt pathway, such as mTOR, GSK-3β, and FOXO3a. This requires more in-depth investigation in future studies.

In conclusion, our study is the first to report a significant protective effect of TUG-891 against neuronal injury induced by cerebral ischemia. This protective mechanism potentially arises from coordinated attenuation of proteotoxic perturbations in the ER, orchestrated through PI3K lipid kinase activity-dependent Akt phosphorylation cascades that recalibrate apoptotic threshold determinants. Consequently, these findings indicate that TUG-891 could serve as a promising novel therapeutic agent for the management of ischemic stroke.

## Author contributions

Dongsheng Fan, Yong Chen and Lili Wei designed research; Panxi Sun, Lili Wei, Xu Qin, Jia Luo performed research; Panxi Sun, Lili Wei analyzed data; Dongsheng Fan and Yong Chen wrote the paper. All authors read and approved the final version of the manuscript.

## Funding Sources

This research was funded by Peking University Medicine Sailing Program for Young Scholars’ Scientific & Technological Innovation (BMU2021PYB040) to Yong Chen; National Natural Science Foundation of China (81873784, 82071426) and Key Project of Peking University Third Hospital (BYSYZD2021004) to Dongsheng Fan; Bingtuan Science and Technology Program (2023ZD040) and Project of Shihezi University (GJHZ202211) to Lili Wei.

## Declaration of Competing Interest

The authors have no relevant financial or non-financial interests to disclose.
